# The influence of load carrying on the energetics and kinematics of terrestrial locomotion in a diving bird

**DOI:** 10.1242/bio.20135538

**Published:** 2013-09-26

**Authors:** Peter G. Tickle, Samantha C. Lean, Kayleigh A. R. Rose, Avanti P. Wadugodapitiya, Jonathan R. Codd

**Affiliations:** Faculty of Life Sciences, University of Manchester, Manchester M13 9PT, UK

**Keywords:** Bird, Diving, Load bearing, Locomotion, Respirometry

## Abstract

The application of artificial loads to mammals and birds has been used to provide insight into the mechanics and energetic cost of terrestrial locomotion. However, only two species of bird have previously been used in loading experiments, the cursorial guinea fowl (*Numida meleagris*) and the locomotor-generalist barnacle goose (*Branta leucopsis*). Here, using respirometry and treadmill locomotion, we investigate the energetic cost of carrying trunk loads in a diving bird, the tufted duck (*Aythya fuligula*). Attachment of back loads equivalent to 10% and 20% of body mass increased the metabolic rate during locomotion (7.94% and 15.92%, respectively) while sternal loads of 5% and 10% had a greater proportional effect than the back loads (metabolic rate increased by 7.19% and 13.99%, respectively). No effect on locomotor kinematics was detected during any load carrying experiments. These results concur with previous reports of load carrying economy in birds, in that there is a less than proportional relationship between increasing load and metabolic rate (found previously in guinea fowl), while application of sternal loads causes an approximate doubling of metabolic rate compared to back loads (reported in an earlier study of barnacle geese). The increase in cost when carrying sternal loads may result from having to move this extra mass dorso-ventrally during respiration. Disparity in load carrying economy between species may arise from anatomical and physiological adaptations to different forms of locomotion, such as the varying uncinate process morphology and hindlimb tendon development in goose, guinea fowl and duck.

## Introduction

Birds adapted to different forms of locomotion exhibit morphological variation. This diversity in body form must be taken into account when examining the factors affecting locomotion and respiration. Despite the fundamental importance of animal locomotion to evolutionary fitness (Tolkamp et al., 2002) the biological mechanisms that account for the energetic costs of terrestrial locomotion are not well understood. Load carrying is a technique that has been used to examine the factors that determine the energetic cost of locomotion. By quantifying the metabolic cost and kinematics of locomotion under unloaded and loaded conditions we can estimate the energetic requirements of functions such as those required to produce the muscular force and mechanical work to move the limbs in birds (Marsh et al., 2006; McGowan et al., 2006; Tickle et al., 2010) and mammals (Steudel, 1990; Taylor et al., 1980). Based upon the directly proportional relationship between increasing load and oxygen consumption found in mammals, Taylor and colleagues suggested that the main factor determining energy consumption during locomotion is the production of muscle force to support body weight (Taylor et al., 1980). More recent research indicates that the metabolic cost of locomotion is made up of a number of components. In addition to the generation of muscle force to support body weight when the foot is in contact with the ground (Taylor et al., 1980), there is an important energetic cost associated with active muscular movement of the ‘swing’ limb during a stride (Ellerby et al., 2005; Ellerby and Marsh, 2006; Marsh et al., 2004). Load carrying studies have produced further evidence for the significant energetic cost incurred when moving the swing limb during locomotion (Marsh et al., 2006; Steudel, 1990; Tickle et al., 2010), in contrast to earlier work which assumed that the process was energetically negligible (Taylor et al., 1980).

In birds the effect of load carriage during terrestrial locomotion has been studied in only two species; guinea fowl, *Numida meleagris*, (Marsh et al., 2006; McGowan et al., 2006) and the barnacle goose, *Branta leucopsis* (Tickle et al., 2010). Guinea fowl are able to carry back loads more economically than the barnacle goose, while the metabolic cost incurred by both species is more economical than the seen in an equivalent sized mammal (Marsh et al., 2006; McGowan et al., 2006; Tickle et al., 2010). Birds and mammals have a similar scaling relationship between the metabolic cost of unloaded locomotion and body size (Taylor et al., 1982), however birds may have novel anatomical, postural and kinematic adaptations that account for their ability to carry loads efficiently (Marsh et al., 2006). Loading studies on birds have the potential to provide new insight as the anatomical adaptations required for the different forms of locomotion (walking, running, swimming, flying or diving) may highlight the underlying factors that determine energetic costs of locomotion. For example, efficient energy storage in the specialised hind limb muscle and tendon units in the cursorial guinea fowl could account for a proportion of the reduced metabolic rate during locomotion when loaded relative to the barnacle goose (Biewener and Corning, 2001; Daley and Biewener, 2003). In contrast, barnacle geese display adaptations for flight and swimming that are not optimal for terrestrial locomotion, such as large flight musculature and webbed feet. Furthermore, drag caused by the body and the feet during swimming may be minimised by placing the legs far back on the body in the goose and other water birds (Zeffer et al., 2003); however moving the legs away from the centre of mass is likely to be sub-optimal for terrestrial locomotion. The trade-off between flight, swimming and terrestrial performance in geese and other semi-aquatic species is reflected in their trunk and limb morphology, waddling motion, restricted gait selection and consequent lower top speed of terrestrial locomotion (Biewener and Corning, 2001; Nudds et al., 2010; Provini et al., 2012; Usherwood et al., 2008). Diving species have an elongated and streamlined body, and powerful leg and/or flight muscles, depending on whether they are wing (e.g. penguins, Sphenisciformes; auks, Alcidae) or foot propelled divers (e.g. diving ducks, Aythyinae; grebes, Podicepiformes). While research into the influence of load bearing has been conducted on two avian species (guinea fowl and barnacle goose) there are no studies using diving species.

The mechanics of level terrestrial locomotion have however been studied in penguins (Baudinette and Gill, 1985; Bevan et al., 1995; Griffin and Kram, 2000; Pinshow et al., 1977), eider duck, *Somateria mollisima* (Hawkins et al., 2000) and cormorant, *Phalacrocorax carbo* (White et al., 2008). When compared to animals with a similar body mass, walking in penguins is expensive in terms of energy consumption (Pinshow et al., 1977). Side-to-side waddling motions, which are common to diving species due to their relatively short and caudally placed legs (Zeffer et al., 2003), were thought to account for the high energetic cost of locomotion (Pinshow et al., 1977). An increase in energetic cost is also thought to relate to the energy consumption of less economical muscle fibres in the penguin leg that must generate a relatively fast rate of force production as a consequence of short hind limbs (Griffin and Kram, 2000). However the cost of waddling is controversial with recent research suggesting that lateral movements of the body in fact enable recovery of mechanical energy over the stride (Griffin and Kram, 2000). Interestingly, the great cormorant (*Phalacrocorax carbo*) is another diving species that has short legs and a pronounced waddling gait, but the energetic cost of terrestrial locomotion does not differ from species adapted to a cursorial lifestyle (White et al., 2008). It is seems that a complicated interaction of factors, which are not yet fully understood, determines the energetic cost of walking in birds.

In addition to adaptations in hind limb structure, avian musculoskeletal respiratory structures vary with specialisation to different forms of locomotion (Tickle et al., 2009; Tickle et al., 2007). When compared to cursorial and generalist birds, diving birds have relatively elongated vertebral and sternal ribs, uncinate processes and sternum (Tickle et al., 2009; Tickle et al., 2007). Birds have a highly derived respiratory system that is comprised of a rigid lung and compliant air sacs (Powell, 2000). Unidirectional airflow across the lungs is accomplished by the coordinated bellows-like action of air sacs (Brackenbury, 1972; Brackenbury, 1973), driven by hypaxial musculature (Codd et al., 2005). Dorso-ventral excursions of the elongate and keeled sternum represent the primary mechanism of generating inspiratory and expiratory airflow in standing birds (Claessens, 2009; Zimmer, 1935). Lateral flaring of the ribs functions as the ventilatory skeletal pump when sternal movement is restricted during sitting (Claessens, 2009; Codd et al., 2005). Flight muscles associated with the sternum account for a large proportion of body mass, up to 35% in some cases (Dial et al., 1988). It is possible that moving this heavy mass during breathing accounts for a significant proportion of energy consumption (Tickle et al., 2010). Artificially increasing the mass of the breast by application of loads up to 15% of body mass in the barnacle goose indicated that moving a heavy sternum is an energetically expensive mechanism relative to an equivalent load carried on the back, causing an approximate doubling in metabolic rate (Tickle et al., 2010). In addition barnacle geese derive energetic savings from changing posture; resting metabolic rate in standing geese is 25% higher than when sitting which is also thought to be driven by the increased cost of standing with relatively heavy breast musculature (Tickle et al., 2012).

Considering the broad differences in locomotor and breathing mechanics across bird taxa (Tickle et al., 2009; Tickle et al., 2007), a larger sample of species is required in order to understand the factors that may affect the energetic cost of ventilation and locomotion. Here we present data on the metabolic cost of locomotion while carrying back and sternal loads in the tufted duck, *Aythya fuligula*, an Anseriforme diving species.

## Results

### Back loading

Attachment of back loads increased the metabolic cost of locomotion. Relative to unloaded locomotion, loads of 10% and 20% precipitated an increase in net metabolic rate of 7.94±3.84% and 15.92±3.67%, respectively (Table 1). General linear modelling (GLM) showed that no effect of load upon resting metabolic rate was found (Table 2). When controlling for the effects of body mass and duck identity, increasing load caused an increase in net metabolic rate (Table 2). Duck identity and body mass, rather than loading condition explained most of the variance in kinematic parameters during locomotion (Table 3).

**Table 1.**
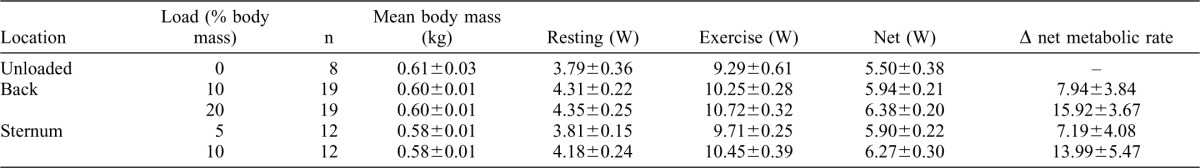
Summary data for the load carrying experiments displaying mean values ± standard error. Number of individual experimental trials for each loading condition is denoted by ‘n’. Metabolic rate has been converted to metabolic power (W) measurement. Net metabolic rate is the difference between exercise and resting rates. Δ net metabolic rate is the % difference in loaded net rate relative to unloaded net metabolic rate.

**Table 2.**
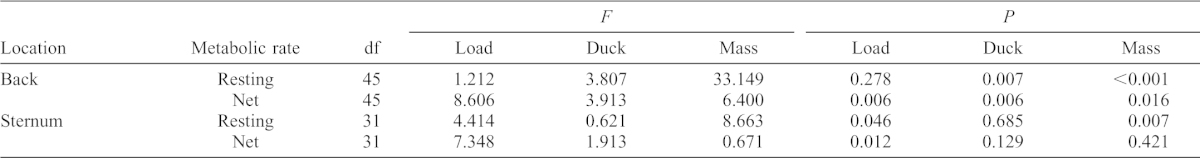
The effects of load carrying, bird identity and body mass on resting and net metabolic rate. Back loads increased net metabolic rate but had no effect on resting metabolic rate. Carrying sternal loads increased resting and net metabolic rate.

**Table 3.**
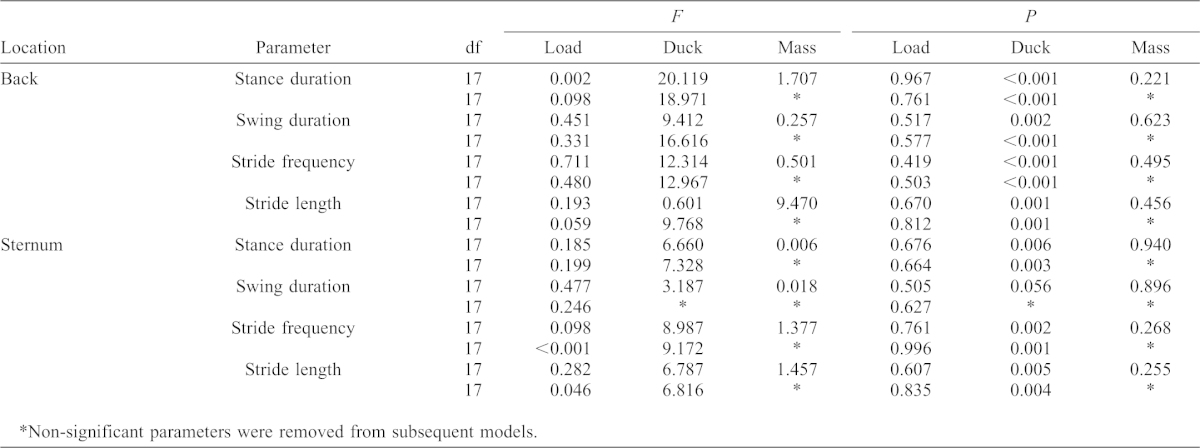
Results of GLM to partition the variation in kinematic parameters due to increasing load, bird identity and body mass. Adding trunk loads did not affect the kinematics of locomotion.

### Sternal loading

Addition of loads to the sternum coincided with a significant increase in the rate of resting and net metabolism. Loads of 5% and 10% were associated with a 7.19±4.08% and 13.99±5.47% increase in net metabolic rate (Table 1). Therefore artificially loading the sternum caused the net metabolic rate to increase by almost double the comparable back loading rate. After accounting for variation caused by identity and body mass using GLM, net rate was significantly affected by increasing size of sternal load (Table 2). After accounting for variation caused by identity and body mass using GLM, net rate was significantly affected by increasing size of sternal load (Table 2). Resting metabolic rate was also significantly increased by application of sternal loads (Table 2); a 10% load caused an increase in the resting rate of 10.31±6.31% (Table 1) compared to the unloaded resting value. No significant effect of loading condition on kinematic parameters during exercise was detected (Table 3).

## Discussion

Here we present the first study into the energetics of load carrying in a bird species anatomically and physiologically adapted to diving. In general the response to load carrying in the tufted duck was similar to previous findings in guinea fowl (Marsh et al., 2006; McGowan et al., 2006) and barnacle geese (Tickle et al., 2010). In tufted ducks the metabolic rate increase during locomotion while carrying back loads was similar to the guinea fowl indicating that there was a less than proportional relationship (around 0.8:1) between the increase in energy consumption and mass of the carried load. Conversely the barnacle goose has a directly proportional, or less economical, relationship between increasing metabolic rate and load (Tickle et al., 2010). Despite this difference, when compared to most mammalian bipeds and quadrupeds (Griffin et al., 2003; Taylor et al., 1980) birds carry loads with greater energetic economy. There are examples of exceptional load carrying economy in a small selection of mammals. Considerable loads can be carried with greater economy compared to control subjects by Nepalese porters (equivalent to a maximum of 173% of body mass) (Bastien et al., 2005) and Luo and Kikuyu tribeswomen (equivalent to 70% of body mass) (Maloiy et al., 1986). The mechanisms that allow for this economic load carriage are not understood, but may include anatomical changes and improved overall efficiency of locomotion (Bastien et al., 2005; Maloiy et al., 1986). In addition, superior storage of elastic strain energy in the hind limb tendons of tammar wallabies means that they can carry loads equivalent to 15% of body mass with no increase in their metabolic rate during locomotion (Baudinette and Biewener, 1998; Biewener et al., 1998).

The barnacle goose and tufted duck are both Anseriform birds meaning that any differences between these closely related species, such as the relatively inexpensive load carriage in the tufted duck, are likely to be caused by specific locomotor adaptations. The divergence in locomotor specialisation between these species may have elicited functional changes in the dynamics of hind limb muscle function. Whilst these data are not available for barnacle geese, elastic energy recovery in the gastrocnemius tendon has been found to be poor in mallard ducks (Biewener and Corning, 2001), representing a considerably smaller proportion of muscle work during locomotion than that found in the tendon of the cursorial turkey (Roberts et al., 1997). Mallard leg movement during walking is primarily powered by muscle shortening rather than isometric contraction coupled with stretch and recoil of tendons as found in running wild turkeys (Roberts et al., 1997). Therefore tendon springs in the duck hind limb are not optimised to reduce the metabolic cost of producing force during terrestrial locomotion. While the barnacle goose is similar to the mallard in terms of morphology, semi-aquatic lifestyle and proficiency at walking, swimming and flying, the effect of tendon energy storage and muscle mechanics on metabolic cost of locomotion are not known. However, it is likely that these species share similar aspects of hind limb mechanics, not least because they both have relatively short legs and thick tendons (Biewener and Corning, 2001; Nudds et al., 2010). Similarly, we speculate that the tufted duck may exhibit specific adaptations in muscle and tendon performance for efficient foot-propelled underwater locomotion that may have an influence on enabling efficient terrestrial load carriage. Clearly, further research is required to elaborate the potential differences in locomotor mechanics between birds adapted to different forms of locomotion.

As in the case of back loading, tufted ducks in the present study were able to carry sternal loads more economically than the barnacle goose; the relationship between increasing metabolic rate and magnitude of sternal load in the barnacle goose is approximately 2:1 (Tickle et al., 2010) while the comparable relationship in the tufted duck is around 1.4:1. Interestingly, the overall energetic cost of walking with a sternal load was approximately double the equivalent back load, just as was found in the barnacle goose (Tickle et al., 2010). These loads may affect the cost of locomotion by increasing the force exerted against the ground by the bird (Taylor et al., 1980) and are comparable when locomotor kinematics are similar (Tickle et al., 2010). No significant changes in the kinematic parameters of locomotion were detected during either back or sternal loading, corresponding to earlier experiments (Marsh et al., 2006; Tickle et al., 2010), indicating that, at least in part, disparity between metabolic rate increase in back and sternally loaded birds may be accounted for by non-locomotor factors (Tickle et al., 2010).

Natural variation in the mass of flight muscles may be relevant to the elevated metabolic rate when carrying sternal loads. Pre-migratory hypertrophy of flight muscles commonly occurs in waterfowl (Ankney, 1979), and this is true in the tufted duck and barnacle goose (Butler and Turner, 1988; Butler et al., 1998). There is a strong similarity between the reported values of flight muscle mass as a proportion of body mass in the tufted duck (16.9–18.5%) (Stephenson et al., 1989; Turner and Butler, 1988) and the barnacle goose (17.6–17.8%) (Butler et al., 1998). Therefore we might expect a similar energetic cost of respiratory caudo-dorsal movements of the sternum. In light of differences in skeletal morphology, however, this may not be the case. The morphology of the uncinate processes varies according to primary locomotor mode; processes are shortest in walking birds, intermediate in non-specialists (birds capable of walking, flying and swimming) and longest in diving species (Tickle et al., 2009; Tickle et al., 2007). Uncinate process length is a factor in the amount of leverage that it can provide for movement of the ribs and sternum during breathing (Tickle et al., 2007). Longer processes in diving species are presumed to result from the general streamlining of the body form to reduce drag during underwater locomotion (Tickle et al., 2009; Tickle et al., 2007). Based on the measurements of Tickle et al. (supplementary material in Tickle et al., 2007), after accounting for body size the tufted duck has uncinate processes over twice the length of those in the barnacle goose. The presumed increase in leverage provided by these elongated processes may be a mechanism by which the duck can offset the energetic cost of carrying artificial mass on the sternum. By reducing the muscle force required to move the ribs and sternum during breathing the greater leverage of longer processes may account for a proportion of the difference in overall metabolic rate between the barnacle goose and tufted duck.

Compared to the barnacle goose the tufted duck has a more elongated body with flight musculature distributed over a longer sternum, likely resulting in a more cranial location of the centre of mass. Application of trunk loads will shift the centre of mass, caudally in the case of sternal loads and dorsally when applied to the back (Tickle et al., 2010). Unknown effects on roll and pitch stability may therefore be incurred, affecting underlying postural and locomotor muscle activity that in turn could account for a proportion of the increased metabolic cost. Consequent changes in the magnitude of body roll associated with the typical waddling gait of Anseriformes, while not quantified in this study, may represent an important factor to explain the variation between back and sternally loaded birds. Interpretation of load carrying studies is confounded by our limited knowledge of these underlying factors but they remain a useful tool to form a basic idea of the overall metabolic costs of various mechanical functions.

Interestingly, resting metabolic rate was increased by application of a sternal load in the tufted duck. This contrasts with the earlier study of barnacle geese where no change in energy consumption was detected, although it appears likely that behavioural changes (resting barnacle geese were observed to sit when carrying a sternal load) may offset the metabolic cost of the load (Tickle et al., 2010). A recent study of resting metabolic rate in unloaded barnacle geese found that when compared to standing, sitting is 25% cheaper in terms of energy expenditure, potentially due to a reduction in the cost of breathing when the sternum is immobile during sitting (Tickle et al., 2012). While an attempt to quantify the energetic cost of avian breathing indicated that it accounts for only 2% of whole-animal metabolism (Markley and Carrier, 2010), our metabolic measurements in resting tufted ducks may suggest otherwise. Maintaining standing posture by isometric contraction of leg muscles is one factor to consider when partitioning the energy cost of resting (Tickle et al., 2012). When we consider the non-significant effect of back loads on resting metabolic rate, it would appear unlikely that increased muscle activity to maintain a standing posture had a considerable effect in sternally loaded birds. Rather, stressing the respiratory system with sternal loads may cause increased ventilatory muscle work to effect rib and sternal movements. In this way the rise in metabolic rate could be accounted for by an increase in respiratory muscle activity and/or recruitment of hypaxial and abdominal accessory breathing muscles (Tickle et al., 2010). Although further research is necessary to test this hypothesis, an earlier study (Codd et al., 2005) has demonstrated the inherent plasticity of the avian respiratory system. Load carrying studies such as those utilised in this study are a useful tool to help decipher the hidden complexities of the musculoskeletal system. While the ultimate energetic costs of breathing and locomotion remain uncertain, experimental manipulation by load carrying provides a novel insight and helps to generate future research objectives.

## Materials and Methods

### Animals

Adult tufted ducks (*Aythya fuligula*) (4 female and 2 male) were obtained from a local supplier and housed together indoors on a 12:12 L: D cycle. Provision of a freshwater pool enabled the birds to exhibit natural diving behaviour. Food (Marine Duck Food; Charnwood Milling Company Ltd., Suffolk, UK: protein 21%, fat 4.396%, ash 5.896%, methionine 0.45%) and water were provided *ad libitum*.

Mean body mass over the duration of the experimental period was 0.58 kg (range: 0.52–0.70 kg). All experiments were approved by the University of Manchester Ethics Committee and conducted in accordance with the Animals (Scientific Procedures) Act (1986) under a UK Home Office project licence held by Dr J. Codd (40/3001).

### Training

Ducks walked on a treadmill (Tunturi T60, Turku, Finland) inside a Perspex® respirometry chamber at a range of speeds (0.14–0.69 ms^−1^). Birds were acclimatised to experimental conditions including treadmill walking and load carrying training for 8 weeks prior to data collection. Unloaded birds were found to walk comfortably for over 10 minutes at 0.42 ms^−1^, which was then used as the experimental speed for subsequent loading trials. As these birds live in social groups a mirror facing the exercising bird together with companion bird sitting next to the treadmill were used throughout all experiments.

### Load attachment

Back and sternal lead loads were adjusted as required before each trial according to individual body mass. Back loads of 10% and 20% or sternal loads equivalent to 5% and 10% of body mass were used during each experiment. Small pieces of duct tape were used to affix loads to the contour feathers on the back or breast, approximately above and below the centre of mass. Loads were attached in such a way so as not to restrict normal breathing movements during standing and sitting.

### Respirometry

Oxygen consumption (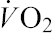) and carbon dioxide production (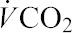) were measured in resting and exercising ducks using open-flow respirometry. Air was pulled through the respirometry chamber (volume: 61 L) at a rate of 48 L min^−1^ using a vacuum pump (Model: 2750CGH160; Thomas, Sheboygan, WI, USA). A sub-sample of excurrent air was removed at 2.5 L min^−1^ from the main pipe exiting the chamber and directed into a bottle. From this bottle, a final sub-sample at 0.1 L min^−1^ was taken using a SS3 pump (Sable Systems, Las Vegas, NV, USA). Water vapour content of excurrent air was measured with an RH-300 humidity meter (Sable Systems, Las Vegas, NV, USA). The air sample was then dried by passing through a magnesium perchlorate column (Acros Organics, NJ, USA). CO_2_ content was then measured using a CA-10a analyser (Sable Systems, Las Vegas, NV, USA) before removal of CO_2_ by passing the airstream through a column of soda lime (Merck, Darmstadt, Germany). Finally the O_2_ content of dry, CO_2_-free air was measured using an Oxzilla II dual channel O_2_ analyser (Sable Systems, Las Vegas, NV, USA). O_2_ content of a parallel sample of scrubbed room air was measured concurrently in a separate channel allowing accurate calculation of 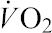. Drift in the O_2_ and CO_2_ channels was removed by transforming the trace in ExpeData® software (Sable Systems, Las Vegas, NV, USA) according to baseline values recorded before and after each trial. Voltage outputs were recorded using a UI2 interface and ExpeData version 1.25 (both Sable Systems, Las Vegas, NV, USA). Introducing a known flow-rate of nitrogen into the empty respirometry chamber allowed for detection of leaks in the gas analysis system; the respirometry apparatus was found to be accurate to 5%, similar to earlier reports (Tickle et al., 2010; White et al., 2008).

As water was removed from the air stream prior to O_2_ and CO_2_ measurement the primary flow rate (FR) was adjusted to a dry-corrected flow rate (FR_c_) using equation 8.6 in Lighton (Lighton, 2008):
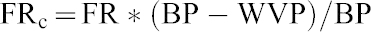
(1)where BP is barometric pressure and WVP is water vapour pressure. 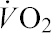 and 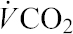 were calculated from equations in Lighton (Lighton, 2008):
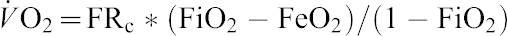
(2)where FiO_2_ is the fractional concentration of O_2_ flowing into the respirometry chamber and FeO_2_ is the O_2_ concentration in air leaving the chamber following removal of water and CO_2_.
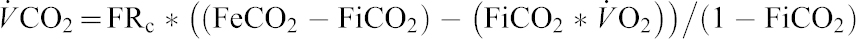
(3)where FiCO_2_ and FeCO_2_ are the fractional concentrations of CO_2_ entering and leaving the respirometry chamber, respectively.

The respiratory exchange ratio (RER) (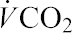: 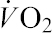) was calculated for each trial. 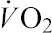 was subsequently converted into metabolic power (W) using the calculated RER values and corresponding thermal equivalents taken from table 12.1 of Brody (Brody, 1945). In each trial resting metabolic rate was subtracted from the total metabolic rate measured during treadmill walking to yield a net metabolic rate. Use of net metabolic rate has been justified in previous loading studies (Marsh et al., 2006; Tickle et al., 2010) on the basis that there is an insignificant change in blood flow to tissues that are not directly involved in locomotion from rest to exercise (Ellerby et al., 2005). Finally, for each loaded trial, the fractional change in metabolic rate was determined as the product of loaded net rate and mean unloaded net rate.

The protocol for data collection followed the methods used by Tickle et al. (Tickle et al., 2010). In brief, pairs of ducks (one experimental and one companion) were chosen at random and a load was attached to the experimental bird. This bird was allowed to walk into the respirometry chamber when a resting trace was measured until stable 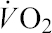 was observed, at which point the treadmill was started. The duck was exercised at a fixed speed (0.42 ms^−1^) until 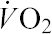 remained steady for around 4–5 minutes. After the treadmill was stopped, resting metabolic rate was measured until the O_2_ and CO_2_ traces returned to a steady level. Baseline fractions of ambient O_2_ and CO_2_ were recorded before and after experiments so that any change in the composition of room-air could be corrected post-trial. Metabolic rate during quiet rest and steady locomotion was derived in each case by using ExpeData® software to select the 60-second plateau with least variability. Mean temperature (± standard error) in the respirometry chamber was 22.17±0.04°C.

### Kinematics

The effects of load upon gait kinematic parameters (stride length, stride frequency, stance phase, and swing phase) were quantified by analysis of footage taken at 100 frames per second using a Sony HDR-XR520 video camera. The position of the left foot was tracked for between 8 and 30 strides during steady locomotion using Tracker 3.10 software (Open Source Physics).

### Statistics

Variation in resting and net metabolic rate was apportioned using general linear modelling (GLM) between duck identity (fixed factor), body mass and load mass (covariates). When load mass had no effect on metabolic rate, another model was built whereby other non-significant parameters were removed on the basis that these factors still account for a small proportion of variation, potentially obscuring a positive result. All statistical analyses were completed in SPSS (SPSS v.19; SPSS Ltd, Chicago, IL, USA). Values are presented as means ± standard error.

## Funding

This research was funded by the BBSRC [BB/011338/1 and BBI021116/1] and the Leverhulme Trust [F/00 120/BH]. P.G.T. was supported by a BBSRC PhD DTA studentship.
